# Airway problems and changing Mallampati score during pregnancy and labor: a systematic review

**DOI:** 10.1186/s44158-025-00279-2

**Published:** 2025-11-18

**Authors:** Maria Giovanna Vastarella, Dario Gaetano, Rossella Molitierno, Nicola Iavazzo, Gianluca Esposito, Luca Gregorio Giaccari, Vincenzo Pota, Pasquale De Franciscis, Pasquale Sansone, Marco La Verde

**Affiliations:** 1 Department of Woman Child and General and Specialized Surgery, University of Campania “Luigi Vanvitelli”, Naples, Italy; 2 Department of Anesthesia and Intensive Care, AO ‘G. Rummo’, Benevento, Italy

**Keywords:** Airway changes, Pregnancy, Delivery, Post-partum, Anaesthesia, Mallampati score, Mallampati class, Mallampati grading

## Abstract

**Background:**

During pregnancy, labor, and postpartum, physiological changes such as weight gain, hormonal fluctuations, or fluid retention may cause airway edema and soft tissue swelling. These factors impact Mallampati Classification, a predictive tool to investigate airway. A higher Mallampati class indicates a high-risk obstetric population. This systematic review explores the airway and Mallampati modification during the different pregnancy periods. Understanding the airway changes in pregnancy is critical to safely intubate, avoid adverse outcomes in maternal care and support clinical practice.

**Methodology:**

Research started using five databases, from the beginning to January 2025: Medline, Embase, Scopus, Cochrane Central Register of Controlled Trials, and ClinicalTrial.gov, with the following search terms: “Mallampati”, “Mallampati grading”, “Airway”, “Airway changes”, “Airway Obstruction”, “pregnancy”, “Delivery, Obstetric”, “Anesthesia, Obstetrical”. Articles included only studies covering a change in the Mallampati classification in pregnancy, labor, or postpartum. All the studies that did not concern airway changes in pregnancy or did not include a Mallampati classification were excluded. The quality of the included studies was assessed using a modified Newcastle–Ottawa Scale.

**Results:**

Ten articles met the inclusion criteria. Significant changes in Mallampati class during pregnancy, labor, and post-partum were evidenced. Grades III and IV of Mallampati Classification increased during pregnancy, reaching 51.7% at delivery, compared to 10.3% pre-pregnancy. Increased proportions of MMC III/IV were observed among women with hypertensive and preeclampsia conditions when compared with the normotensive population. During the postpartum, the Mallampati class partially receded toward pre-pregnancy levels, with approximately 82% returning to baseline within 36 to 48 h.

**Conclusion:**

These findings evidenced the pregnancy-related airway changes and their dynamic process, highlighting the need for close vigilance, especially during labor in high-risk populations.

**Systematic review registration:**

PROSPERO CRD42025635304.

**Supplementary Information:**

The online version contains supplementary material available at 10.1186/s44158-025-00279-2.

## Introduction

The Mallampati classification (MCC) which was proposed in 1985 is a widely accepted tool for assessing airway based on oropharyngeal visibility [1]. It categorizes patients into four classes I, II, III, and IV, with higher classes indicating increased difficulty in visualizing the vocal cords during intubation, thus elevating the risk during the procedure [2]. This system is important in pre-anesthetic assessments as it helps identify potential complications in airway management [3]. Invasive approaches in gynecological patients emphasize the importance of preoperative planning and tailored approaches for detecting problematic cases and optimizing the outcomes [4–6]. MCC represents the most common system employed for the prediction of airway difficulty and assumes great importance in obstetric patients due to physiological and anatomical changes during pregnancy and labor [7]. The increasing inductions of labor and analgesic procedures have increased the pre-labor anesthetic evaluation [8–10]. However, few studies have explored the direct relevance of this classification change in obstetrics. A progressive MCC modification appears related to hormonal changes, increased vascular engorgement, and fluid retention, further exacerbated during labor by stress-induced edema and fluid shifts [11]. Studies confirm a significant increase in the percentage of patients with Mallampati classes III or IV from pre-pregnancy to delivery [12]. Such scores are highly correlated with increased risks of difficult laryngoscopy and intubation [13]. Some studies showed that 20–34% of pregnant women experience a worsening MCC during labor compared to their baseline [14]. Additional debates remain on diagnosing these changes and on conditions such as preeclampsia that further worsen airway narrowing. Gestational hypertensive disorders demonstrate a significantly higher propensity for elevated MCC as compared to normotensive counterparts [15]. Understanding of dynamic changes in airway during pregnancy is vital for anesthesiologists and healthcare providers managing obstetric patients because failure to anticipate and adapt to these challenges can lead to serious complications such as failed intubation and hypoxia during emergency cesarean sections [12]. The dynamic changes of pregnancy are related to hormonal influences, including vascular engorgement and mucosal edema, with changes in the upper respiratory tract [12]. Weight gain and fluid retention represent additional mechanical factors to airway narrowing and MCC modification [16]. In addition, during labor, fluid shifts and stress-induced edema worsen airway management [17]. These factors emphasize the need for a detailed assessment of airway changes in obstetric patients since these factors affect the safety of intubation and the possibility of failed laryngoscopy. Despite the extensive use of the Mallampati score in clinical practice, its specific relevance for airway management in an obstetric setting remains not entirely explored.

This systematic review attempts to summarize those knowledge gaps that act as the background for dynamic variations during pregnancy and labor periods utilizing the Mallampati score. Second, this systematic review explores the impact of the gestational hypertensive condition on airway narrowing. Understanding the dynamic changes in the Mallampati classification could improve obstetric airway management, especially in resource-poor countries.

## Methods

### Eligibility criteria

We included only studies that explore Mallampati score during pregnancy, labor delivery and postpartum. Studies engaged in the research include randomized controlled trials (RCT), observational, cohort studies, and case series of pregnant patients who had airway assessment during the pregnancy. Studies were excluded if they did not assess pregnancy-related airway changes or lacked sufficient data on Mallampati classification. Others are papers drawn from non-pregnant populations or those in which the Mallampati class was not the study focus and are not included. Non-English language papers and case reports were excluded.

Ethics committee approval was not required for this systematic review using only publicly available data from published studies.

### Information sources and search strategy

Our methodology in this systematic review (PROSPERO ID: CRD42025635304) adheres to PRISMA guidelines [18] and is validated by the Enhancing the Quality and Transparency of Health Research (EQUATOR) network and the Cochrane Handbook for Systematic Reviews [19] to ensure transparency and accuracy. A precise search strategy was employed across major databases including electronic databases Medline, Embase, Scopus, Cochrane Central Register of Controlled and Research Register (ClinicalTrial.gov) and our search terms were “Mallampati”, “Mallampati grading”, “Airway”, “Airway changes”, “Airway Obstruction”, “pregnancy”, “Delivery, Obstetric”, “Anesthesia, Obstetrical” (Appendix 1). Our search was not restricted to specific dates and was performed from inception of each database until 01 January 2025.

### Study selection

Two authors (D.G. and N.I.) independently screened the titles and abstracts retrieved by the search strategy for studies included. The same two independent authors (D.G. and N.I.) checked the full texts of the articles possibly eligible for inclusion. This was supplemented with other research findings through a manual search of the reference lists of identified eligible studies. Differences in eligibility assessment were resolved by discussion with a third reviewer (M.L.V.). All authors agreed on the final selection of included studies.

### Data extraction

Two authors (D.G. and N.I.) independently extracted data from the included articles using a pre-piloted standard form about features of the study, characteristics of the populations, stages of gestation, complications, and outcomes; this was cross-checked by a third reviewer (M.L.V.) to ensure that all data were extracted accurately and completely.

### Assessment of risk of bias

We initially planned to use the RoB 2 tool for RCTs, but no RCTs were identified during study selection. Therefore, only the modified Newcastle–Ottawa Scale (mNOS) was used to assess the observational studies. The assessment of the risk of bias for the studies included in this systematic review was performed by two independent reviewers (D.G. and N.I.), using the mNOS [20]. Modified Newcastle–Ottawa scale used for appraising the quality of the study across five important domains: “study design and sample representativeness”, “Sampling technique”, “Description of The Mallampati score application”, “quality of the population description”, and “incomplete outcome data” (Table S1).

### Outcome measures and data synthesis

The main objective of the present review was to establish the evaluation of a Mallampati score change during pregnancy and post-partum. Quantitative analysis was not possible due to the different heterogeneity of outcomes. Although meta-analysis and subgroup analyses were planned according to the PROSPERO protocol (CRD42025635304), these were not conducted due to the variability of reported data. We provide a narrative synthesis instead. We performed a descriptive synthesis of the results according to the type of evaluation, population analyzed, and trimester of pregnancy.

## Results

### Study selection and characteristics

**Fig. 1:**
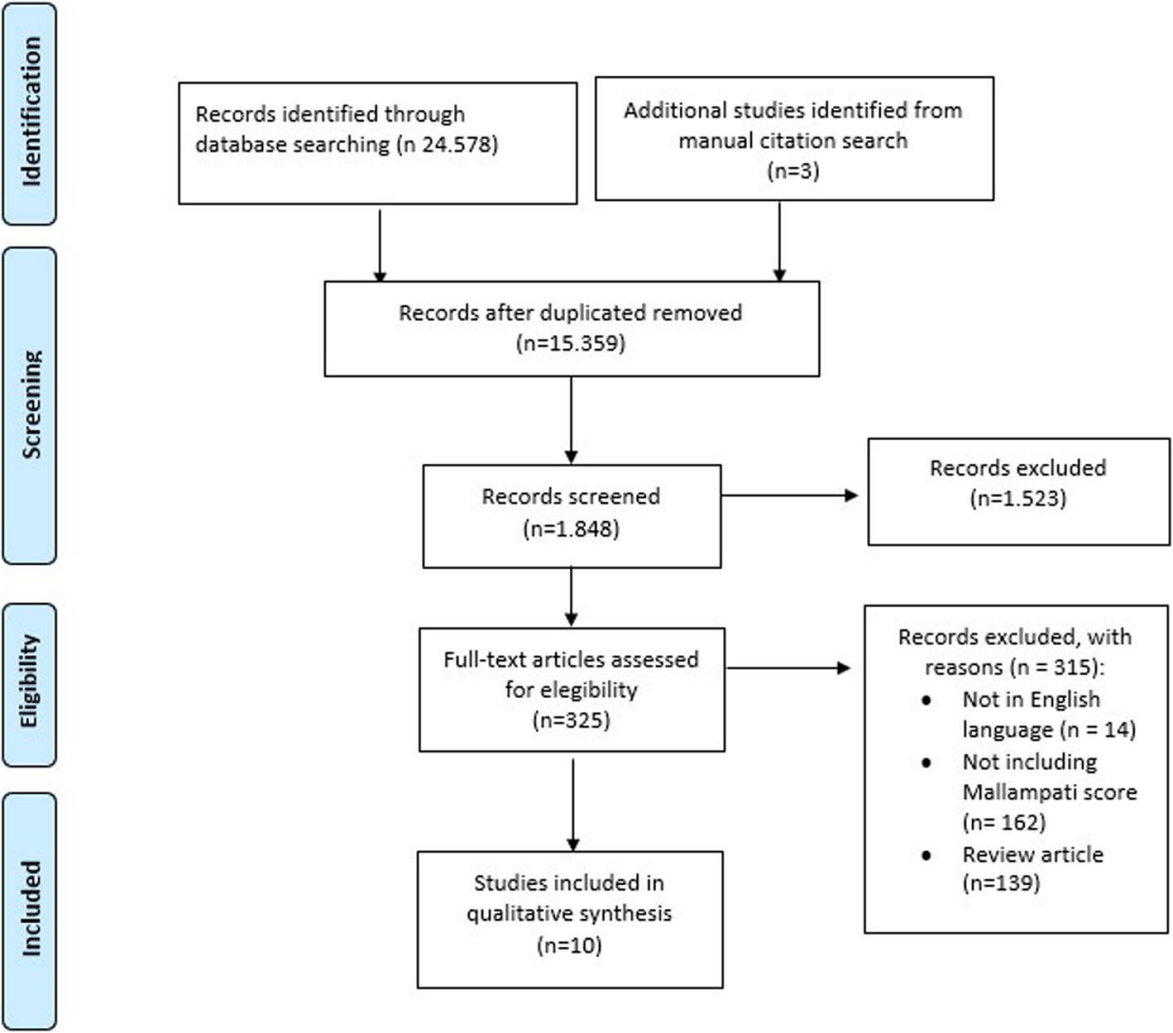
PRISMA flow diagram of study selection

The study selection process is illustrated in Fig. 1.

After the full text evaluation, ten papers that fulfilled the inclusion criteria mentioned above were included in the present systematic review [21–30]. Table 1 shows the main characteristics of the studies included. All the studies included had prospective study designs and were published between 1995 and 2021 [21–28]. All ten studies were prospective observational studies; no randomized controlled trials (RCTs) were identified. The manuscript lacked explicit information about whether these studies were single-center or multicenter [21–30]. Table 1 shows the main characteristics of the studies included, including available data on intervention or exposure groups (e.g., pregnancy stage, labor vs. pre-labor, or presence of preeclampsia) and comparison groups where reported. Of these, four studies were from Europe [21, 23, 27, 29], five from Asia [24–26, 28, 30] and one from America [22]. The number of patients included fluctuates from 61 patients to 242 [21–30].

**Table 1:** Population characteristics

Study, year	No. of patients	Maternal age (years)	BMI	Gestational age (trimester)	Type of delivery (vaginal/cesarean)	Specific population characteristics
Kaur K et al. 2023 [30]	90	> 20	NR	Second trimester, 32–34 weeks, 38–40 weeks	Vaginal, cesarean	–
Aydas AD et al. 2015 [27]	61	17–39	NR	37	Vaginal	–
Bountonnet M et al. 2010	87	31.2 (5.2)	23.9 (4.7)	Third trimester	58 vaginal, 9 caesarean	3 pre-eclampsia, 5 hypertension, 10 diabetes
Guru R. et al. 2013 [29]	190	31.4 (epidural), 32.6 (no epidural)	29.1 (6.0) Epidural 28.7 (7.3) no epidural	40.0 weeks (epidural), 39.0 weeks (no epidural)	NR	–
Pilkington S et al. 1995 [23]	242	NR	NR	NR	NR	–
Ahuja P et al. 2018 [26]	50	NR	NR	Early labor	NR	Preeclampsia (severe in one group)
Bala R et al. 2023 [24]	165 (55 non-pregnant, 55 normotensives pregnant, 55 pre-eclamptic)	NR	NR	NR	NR	Pre-eclampsia (in one group)
Kodali et al. 2014 [31]	61 (first study); 21 (second study)	31–32	NR	NR	Vaginal: 70 Cesarean: 9	–
Sangkum L et al. 2021 [25]	104	31.4 ± 5.1	III 11.6%	Third trimester	Cesarean	Only cesarean section
Raza D et al. 2017 [32]	30 (hypertensive pregnant); 30 (normotensive pregnant)	31.8 ± 3.45 hypertensive pregnant 25.90 ± 4.65 normotensive pregnant	30.67 ± 4.34 29.0 ± 2.98	37–38 weeks	Vaginal: 44 Cesarean: 16	Gestational hypertension in one group

### Risk of bias of included studies

All the studies included were presented with a low risk of bias, five were rated to have a low risk of bias in three domains [21–23, 27, 30], while the remaining five have a low risk of bias in more than three domains [24–26, 28, 29]. A detailed outline of the risk of bias for each domain among the studies is given in Table S2. To evaluate the methodological quality of the included studies, we used a modified Newcastle–Ottawa Scale (mNOS), assessing five domains: study design and sample representativeness, sampling technique, description of the Mallampati classification and evaluation timing, quality of population description, and completeness of outcome data. Overall, the risk of bias was low to moderate across most studies. Three studies (Bala et al. 2023; Sangkum et al. 2021; Ahuja et al. 2017) achieved the highest total scores (≥ 4 stars), indicating relatively stronger methodological quality. Most studies adequately described their sampling techniques and Mallampati assessment procedures, though several lacked complete outcome data or detailed population descriptors. No study received a perfect score across all five domains.

### Synthesis of the results

Sample sizes of the included studies ranged from 20 to 242 participants aged between 17 and 39 years (Table 1) [21–30]. When available, the BMI during pregnancy was different between the different studies [21, 25, 28, 29]. The gestational ages included in the analyses contained all trimesters of pregnancy, particularly the third trimester and pre-delivery weeks (37–40 weeks of pregnancy). Some studies explored specific populations affected by preeclampsia or gestational hypertension [21, 24, 26, 28]. One paper discusses the epidural influence [29]. Table 2 includes MMC changes across pregnancy and post-partum.

**Table 2:** Mallampati classification analysis across different pregnancy stages

Study	Pre-pregnancy (%)	I Trimester (%)	II Trimester (%)	III Trimester (%)	During labor (%)	Postpartum (%)
Kaur K et al	–	–	X	X	X	X
Aydas AD et al	X	X	X	X	X	X
Bountonnet M et al	X	–	–	X	X	X
Raza D et al	–	–	–	–	X	-
Guru R et al	–	–	–	–	X	-
Kodali BS et al	–	–	–	–	X	X
Pilkington S et al	–	X	X	X	–	–
Ahuja P et al	–	–	–	-	X	X
Bala R et al	–	–	–	X	–	–
Sangkum L et al	–	–	–	-	X	X

Notably, eight studies examined MMC during labor [21, 22, 25–30], while others examined the pregnancy stages, first, second, and third trimesters [23, 27]. Six articles analyzed the postpartum regression of the MMCs and established trends in their reversibility post-delivery (Table 2) [21, 22, 25–27, 30]. Several studies have demonstrated an MMC increase during pregnancy and labor. Boutonnet et al. reported increased Mallampati classes 3 and 4 during labor (Table 1) [21]. They showed an incomplete reverse within 48 h of postpartum [21]. Kodali et al. have reported a trend toward higher Mallampati classes from pre- and post-partum periods (Table 1) [22]. Pilkington et al. evidenced an increase of four Mallampati classes after 38 weeks of gestation [23]. Comorbidity conditions (for example, pre-eclampsia) were included (Table 1). Three studies have reported increased airway modifications associated with pre-eclampsia, which suggests a strong link between pre-eclampsia condition and MMC [24, 26, 28]. Postpartum Mallampati score regression was found in different studies, but not all included studies explored the postpartum period (Table 1) [21, 24, 26, 28]. According to Aydas et al. (2014), significant Mallampati scores regress within 24 h following delivery was documented [27]. Two studies evidenced persistent airway change for longer periods post-Caesarean section or complicated delivery [21, 25]. Guru et al. suggested that epidural analgesia did not significantly affect the airway class, and the airway changes occurred independently of epidural interventions (Table 1) [29]. All these findings supported the concept that the Mallampati score can be considered a pregnancy-related physiological change. Moreover, these modifications would remarkably impact airway management, especially in addition to comorbid conditions like pre-eclampsia or emergency cesarean section.

Table 3 is a summary table presenting the key quantitative findings from the included studies.

**Table 3:** Quantitative findings

Study	Timepoints assessed	Key findings
Boutonnet et al., 2010	8th month, epidural, post-delivery (20 m), 48 h	MMC III–IV rose from ~ 10% to 52%, declined to 21% at 48 h
Pilkington et al., 1995 [23]	12 weeks vs 38 weeks	MMC Class IV increased by 34% at 38w (r≈0.3 with weight gain)
Kodali et al., 2008 [22]	Pre and post-delivery	Reported airway narrowing during labor
Ahuja et al., 2018 [26]	Pre-labor to post-labor	MMC increased with preeclampsia from pre- to post-labor
Raza et al., 2018 [28]	Hypertensive vs normotensive in labor	Higher MMC scores in hypertensive women during labor
Guru et al., 2013 [29]	With vs without epidural during labor	Epidural did not significantly affect MMC; changes seen in both groups
Aydas et al., 2015 [27]	Before and after delivery	Significant regression of MMC within 24 h post-delivery
Bala et al., 2023 [24]	Non-pregnant, normotensive, preeclamptic	MMC significantly higher in preeclamptic vs others
Sangkum et al., 2021 [25]	Before and after cesarean	Increased MMC score after cesarean
Kaur et al., 2023 [30]	Second trimester, 32–34w, 38–40w, postpartum	Progressive MMC changes with gestational age

## Discussion

### Mallampati classification and airway management

The Mallampati classification represents a well-established tool in the preoperative assessment of airway management [33]. The score was first proposed in 1985 by Seshagiri Mallampati [34]. The classification involved a subjective evaluation of the oropharyngeal structure, meaning soft palate, uvula, and posterior pharyngeal wall [35]. The clinician evaluated the airway by asking the patient to open the mouth and protrude the tongue [36, 37]. The classification includes four classes. Class I is when the soft palate, uvula, and tonsillar pillars are discernible. In Class II, the uvula and soft palate are partially visible. Class III allows visualization of the soft palate and base of the uvula, while Class IV indicates that only the hard palate is visible. The classes of Mallampati have been related to the risk of airway management risks, such as difficult tracheal intubation or mask ventilation [38]. Higher classes, especially Class III or IV, indicate a higher risk [33, 39]. The Mallampati test supported the choice of airway management strategies and equipment and gave predictive value when combined with other clinical parameters such as neck mobility, thyromental distance, and mouth opening, along with a history of difficult intubation [40]. However, despite its wide application, the Mallampati classification has limitations: inter-observer variability, patient positioning, and compliance [41]. These factors may alter the perceived class. Also, anatomical variations due to obesity, facial trauma, or pathological conditions such as airway edema impact the test evaluation [7]. The Mallampati score should be applied in the setting of a comprehensive clinical assessment associated with other predictors of difficult airway [42].

### Airway and circulatory changes modification in pregnancy

During pregnancy, physiological changes are observed [43]. These changes support fetal development and prepare for delivery [43]. Different factors support the physiologic changes, such as hormonal, mechanical, and metabolic [44]. During pregnancy, maternal blood volume increases progressively and reaches about 40–50% prepregnancy levels during the late third trimester [45]. Such an increase in blood volume consists, to a significant part, of increased plasma volume, thus causing a physiologic dilution of the red blood cell mass and a decline in hematocrit [45]. In the same way, cardiac output increases of 30–50%, mainly in relation to augmented blood volume and pulse rate [46]. Systemic vascular resistance declines secondarily to the vasodilatory properties of progesterone and relaxin, as well as the creation of a low-resistance placental circulation [46, 47]. Cardiac output further increases during labor due to uterine contractions. The respiratory adaptations are no less important [48]. The increased sensitivity of the central respiratory centers to carbon dioxide, carried by progesterone, causes hyperventilation with a 30–40% rise in tidal volume and resultant increase in minute ventilation [49]. Functional residual capacity is reduced due to the expanding uterus elevating the diaphragm, while total lung capacity is relatively preserved [50]. During labor, physical exertion and hyperventilation induced by pain may further alter the respiratory mechanics and acid–base balance, thus requiring careful monitoring of oxygenation and ventilation [51]. The other classic changes during pregnancy are related to renal changes [52, 53]. Essentially, these changes arise through the effects of increased cardiac output and under the influences of hormones, including relaxin [52]. Sodium and water are retained to support the increased plasma volume through stimulation by the renin–angiotensin–aldosterone system, although peripheral vasodilation in normotensive pregnancies prevents significant hypertension [54]. Physiological pregnancies well-tolerated these changes. Moreover, when comorbidities are present, these changes increase the feto-maternal risks and support the obtaining of optimal results with less burden of complications both antenatally and during the intrapartum period [55, 56].

### Mallampati changes in pregnancy and clinical implications

The MCC represents a common easy tool to estimate airway management [57]. As reported by our systematic review, pregnancy related changes significantly impact this score. Several studies have demonstrated progressive increases throughout pregnancy, during labor, and before delivery, reflecting weight gain, hormonal changes, and fluid retention with resultant edema of the pharynx [58, 59]. This change is a dynamic process, an increase in the number of difficult airway cases during labor, followed by partial or complete regression postpartum [58, 59]. There are many studies that reported a high incidence of high classes Mallampati, reflecting difficult airway management, significantly in advanced pregnancy [42]. Boutonnet et al. and Kodali et al. demonstrated that during labor, the Mallampati grade III-IV incidence increased with respect to the pre-labor period [21, 22]​. These changes did not tend to resolve in the postpartum period (48 h after the delivery) [21]​. This dynamic nature underlines the transient influence of pregnancy on airway anatomy but also points to critical periods of increased risk [59]. The number of women with Mallampati III or IV increased by 34% from the first to the third trimester, according to Pilkington et al. [23]​. They evidenced a body weight gain impact on their findings [23]. Bala et al. evidenced a significant difference in Mallampati score between non‐pregnant, normotensive pregnant women, and pregnant women with preeclampsia [24]. Also Ahuja et al. explored the preeclamptic population and evidenced a Mallampati score increase from the pre-labor to the post-labor period [26]. Sangkum et al. explored only cesarean delivery and evidenced a significant increase in MMC score after cesarean delivery [25]. Epidural analgesia and Mallampati scores were explored by Guru et al., with a Mallampati score alteration related to labor in one third [29]. In accordance, Aydas et al. confirmed a Mallampati score change in one-third of the patients 24 h after delivery [27]. These findings highlight the need for reevaluation of the Mallampati scores at various times in pregnancy and especially in association with other comorbidities or at the delivery time. All these findings, clinically demand special consideration in the management of the airway in pregnant women, especially for high-risk patients requiring general anesthesia in labor and delivery [60]. The reevaluation of the airway in late pregnancy and during labor will support the anesthesiologist management. Equipment for advanced airway management by video laryngoscope and fiberoptic intubation should be immediately available, especially for high-risk patients like preeclamptics [61].

This systematic review has several strengths, enhancing the validity and relevance of its findings. A rigorous methodology was applied to different databases. Our focus on dynamic changes in the Mallampati classification during pregnancy, labor, and postpartum gives critical information in airway management in obstetric patients. Despite the strengths, different limitations are present. The included studies presented a high population heterogeneity and different designs. Most of the studies were accomplished on small sample sizes or were not uniform in terms of the assessment of MMC; therefore, this introduces variability into our findings. At least the studies included analyzed different pregnancy periods.

Mallampati changes highlight the necessity of a multidisciplinary collaboration between anesthesiologists and obstetricians. This multidisciplinary approach offers optimal assistance, especially during critical conditions that necessitate of timely intervention. Further studies would still be indicated to clearly elucidate other predictive factors for airway changes in pregnancy. Weight gain and fluid retention are well-recognized contributing factors, individual anatomical variability and effects of epidural anesthesia require further explanation. At least, Bala et al. showed a promising future line in the improvement of the preoperative assessment by ultrasonographic examination for the prediction of a difficult airway [24]. Moreover, additional studies are needed to confirm Mallampati score modification and ultrasonographic parameters.

Finally, it is important to note that the Mallampati classification is no longer considered the most reliable predictor of difficult airways. While the Mallampati classification remains widely used, it is often criticized for its low sensitivity (~ 0.39) despite reasonable specificity (~ 0.86) in predicting difficult intubation. Alternative tools, such as the El Ganzoury combined score and the Upper Lip Bite Test (ULBT), offer greater specificity and may provide better prediction of airway difficulty [62, 63]. The ULBT demonstrates higher diagnostic accuracy—with pooled sensitivity around 0.52 and specificity around 0.84—and in some study settings achieves sensitivity and specificity exceeding 90%. Furthermore, multivariate predictive tools like the El Ganzouri Risk Index (EGRI) combine multiple airway parameters to improve predictive performance, with thresholds ≥ 4 achieving sensitivity ~ 81.6% and specificity ~ 85.5% in recent validation studies. This should be considered in the interpretation of our findings.

## Conclusion

Changes in MMC during pregnancy, labor, and postpartum illustrate dynamic anatomical and physiological shifts which may elevate airway management risks, particularly during labor. Notable increases in MMC are observed in hypertensive and pre-eclamptic women, stressing the needs of tailored airway management strategies.

## Supplementary Information

Supplementary Material 1.

Supplementary Material 2.

Supplementary Material 3.

## Data Availability

No datasets were generated or analysed during the current study.
