# Supplementary material for: Airway problems and changing Mallampati score during pregnancy and labor: a systematic review
Source: J Anesth Analg Crit Care. 2025 Nov 18;5:80. doi: 10.1186/s44158-025-00279-2 (PMC12625491; doi:10.1186/s44158-025-00279-2)
Supplement: Supplementary file 1 — Supplementary Material 1. [file 44158_2025_279_MOESM1_ESM.docx]

**APPENDIX 1**

**Detailed search strategy for systematic review.**

| **Variable** | **Search strategy** |
| --- | --- |
| Database searched | PubMed, EMBASE, Scopus, Google Scholar and Cochrane Central Register of Controlled Trials from inception of each database until 01 January 2025. |
| Search strategy for MEDLINE (accessed through PubMed) | ((((Mallampati) OR (Mallampati grading)) OR (Airway)) OR (Airway changes)) OR (("Airway Obstruction"[Mesh])) AND ((("Pregnancy"[Mesh])) OR ("Delivery, Obstetric"[Mesh])) OR ("Anesthesia, Obstetrical"[Mesh]) |
| Scopus | TITLE-ABS-KEY ((((Mallampati) OR (Mallampati grading)) OR (Airway)) OR (Airway changes)) OR (("Airway Obstruction"[Mesh])) AND ((("Pregnancy"[Mesh])) OR ("Delivery, Obstetric"[Mesh])) OR ("Anesthesia, Obstetrical"[Mesh]) |
| EMBASE | ('mallampati' OR 'mallampati grading' OR 'airway'/exp OR 'airway' OR 'airway changes' OR 'airway obstruction'/exp OR 'airway obstruction') AND ('pregnancy'/exp OR 'pregnancy' OR 'delivery, obstetric'/exp OR 'delivery, obstetric' OR 'anesthesia, obstetrical'/exp OR 'anesthesia, obstetrical') |
| Cochrane Central Register of Controlled Trials | ((((Mallampati) OR (Mallampati grading)) OR (Airway)) OR (Airway changes)) OR (("Airway Obstruction"[Mesh])) AND ((("Pregnancy"[Mesh])) OR ("Delivery, Obstetric"[Mesh])) OR ("Anesthesia, Obstetrical"[Mesh]) in Title Abstract Keyword |
| Research Register  (ClinicalTrial.gov) | Mallampati, Mallampati grading, Airway, Airway changes, Airway Obstruction, Pregnancy, Delivery, Obstetric, Anesthesia, Obstetrical |
| Other sources | The reference lists of selected articles were hand searched to identify any relevant articles. |
