# Supplementary material for: Airway problems and changing Mallampati score during pregnancy and labor: a systematic review
Source: J Anesth Analg Crit Care. 2025 Nov 18;5:80. doi: 10.1186/s44158-025-00279-2 (PMC12625491; doi:10.1186/s44158-025-00279-2)
Supplement: Supplementary file 2 — Supplementary Material 2. [file 44158_2025_279_MOESM2_ESM.docx]

**(1) Study design and sample representativeness:**

1 point: Study design involved a control group, the sample size was greater than or equal to 100.

0 points: Uncontrolled study, sample size less than 100 participants.

**(2) Sampling technique:**

1 point: Patients recruited consecutively or randomly (randomization criteria clarified).

0 points: Potential convenience sampling or unspecified sampling technique.

**(3) Description of the Mallampati and evaluation time:**

1 point: The authors provided a comprehensive description of the equipment, setting, and

adopted technique.

0 points: The study did not report adequate information on the Mallampati evaluation technique.

**(4) Quality of population description:**

1 point: The study reported a clear description of the population with proper measures of dispersion (e.g., mean, standard deviation) and control group description.

0 points: The study did not report a clear description of the population, incompletely reported

descriptive statistics or did not involved a control group**.**

**(5) Incomplete outcome data:**

1 point: The study reported complete data.

0 points: Selective data reporting cannot be excluded.

**Table S1.** Modified Newcastle-Ottawa scoring items.

The individual components listed above are summed to generate a total modified Newcastle-Ottawa

risk of bias score for each study. Total scores range from 0 to 5.

For the total score grouping, studies were judged to be of low risk of bias (≥3 points) or high risk of bias

(<3 points).
