# Supplementary material for: Airway problems and changing Mallampati score during pregnancy and labor: a systematic review
Source: J Anesth Analg Crit Care. 2025 Nov 18;5:80. doi: 10.1186/s44158-025-00279-2 (PMC12625491; doi:10.1186/s44158-025-00279-2)
Supplement: Supplementary file 3 — Supplementary Material 3. [file 44158_2025_279_MOESM3_ESM.docx]

| **Table 2.** Risk of bias assessment of the 9 included studies | | | | | | | |  |  |
| --- | --- | --- | --- | --- | --- | --- | --- | --- | --- |
| Author, Year | **Study design and**  **sample**  **representativeness** | **Sampling**  **technique** | **Description of the Mallampati and evaluation time** | | **Quality of**  **population**  **description** | | **Incomplete**  **outcome**  **data** | | **Total**  **score** |
| Boutonnet M. 2010 | - | ★ | | ★ | | - | ★ | | ★ ★ ★ |
| Kodali B. 2008 | - | ★ | | ★ | | - | ★ | | ★ ★★ |
| Pilkington S. 1995 | ★ | ★ | | ★ | | - | - | | ★★ ★ |
| Bala R. 2023 | ★ | ★ | | ★ | | ★ | ★ | | ★★★ ★ ★ |
| Sangkum L. 2021 | ★ | ★ | | ★ | | - | ★ | | ★ ★ ★★ |
| Ahuja P. 2017 | - | ★ | | ★ | | ★ | ★ | | ★ ★ ★★ |
| Aydas A.D. 2014 | - | ★ | | ★ | | - | ★ | | ★ ★★ |
| Guru R. 2013 | ★ | ★ | | ★ | | - | ★ | | ★ ★★★ |
| Raza D.2018 | - | ★ | | ★ | | ★ | ★ | | ★ ★ ★★ |
| Kaur K. 2023 | - | ★ | | ★ | | - | ★ | | ★ ★ ★ |
|  |  |  | |  | |  |  | |  |
|  | | | | | | | |  |  |
